# Editorial: Insights in orthopaedic surgery: 2021

**DOI:** 10.3389/fsurg.2023.1261106

**Published:** 2023-08-11

**Authors:** Jaimo Ahn

**Affiliations:** Orthopaedic Surgery, Michigan Medicine, University of Michigan, Ann Arbor, PA, United States

**Keywords:** future, evidence, advances, information, knowledge

**Editorial on the Research Topic** Insights in orthopedic surgery: 2021

When we opened up this topic in just past 2020, orthopaedic surgery was moving at a pace and in ways that we could not have predicted even a few decades ago. Certainly, the way we examine, evaluate and investigate our thoughts, behaviors and results continues to change year to year, even month to months. Some of our most spirated debates now incorporate psychosocial nuances of care, honest solutions around preventing opioid-induced deaths and…artificial intelligence Zhou et al. all topics that constituted background noise at the turn of the 21st century. In this context, we thought it would be informative to open up a topic with an open editorial agenda; we asked our thinkers to provide us with a glimpse of orthopaedics today.

What we found was that some issues that have plagued our patients continue to do so although some of the ways we evaluate and treat them have changed dramatically over the years: back pain and symptoms from lumbar disc herniation Wan et al. shoulder dysfunction from rotator cuff tears Zhou et al. and Jin et al. hip fractures and frailty of our older family members Yu et al. and our never ending dance with our microbial co-habitants Kankilic et al. But besides the new knowledge and skills gained over time, the way we analyze what we have done continues to make progress in ways difficult to predict. In addtion to the standard clinical study methodologies, systematic review and meta-analyses, our compendium now includes modalities such as the aforementioned artificial intelligence and modern ways of conceptualizing our research trends and attention through bibliometric analysis, be it for ankle fractures, patellar instability or rotator cuff disease Zeng et al., Zheng et al. and Jin et al.

As we progress through this decade and beyond, what will we see? AI assembled meta-analyses and registry reports? A dashboard that provides live bibliometric data on-demand? A decision aid phone app that is up-to-the-second current on the literature? Wiil the lines between “research” and data-driven patient care blur? What kind of “knowledge” will we be reporting on in 2050? Bone glue that supports weight-bearing, adaptive surgical implants that guide tissue healing, a cell-programming biologic that requires no surgery at all? I am excited to not only find out but to participate and contribute; and hope that you are too.

